# Bufei Jianpi granules improve skeletal muscle and mitochondrial dysfunction in rats with chronic obstructive pulmonary disease

**DOI:** 10.1186/s12906-015-0559-x

**Published:** 2015-03-10

**Authors:** Yuqiong Dong, Ya Li, Yafei Sun, Jing Mao, Fengjia Yao, Yange Tian, Lili Wang, Linlin Li, Suyun Li, Jiansheng Li

**Affiliations:** Dongzhimen Hospital, Beijing University of Chinese Medicine, Beijing, 100700 China; Institute for Respiratory Diseases and the Level Three Laboratory of Respiration Pharmacology of TCM, the First Affiliated Hospital, Henan University of Traditional Chinese Medicine, Zhengzhou, Henan 450000 China; Central Laboratory, the First Affiliated Hospital, Henan University of Traditional Chinese Medicine, Zhengzhou, Henan 450000 China; Collaborative Innovation Center for Respiratory Diseases Diagnostics, Treatment and New Drug Research and Development in Henan Province, Zhengzhou, Henan 450046 China; Institute for Geriatrics, Henan University of Traditional Chinese Medicine, Zhengzhou, Henan 450046 China

**Keywords:** Bufei Jianpi granules, Chronic obstructive pulmonary disease, Skeletal muscle dysfunction, Morphormetry, Mitochondria

## Abstract

**Background:**

Bufei Jianpi granules has been confirmed effective in improving pulmonary function, alleviating acute exacerbations, improving six-minute walk distance and quality of life, and benefited in 12-month follow-up in chronic obstructive pulmonary disease (COPD) patients with syndrome of lung-spleen qi deficiency. Skeletal muscle dysfunction (SMD), an important extrapulmonary complication, occurs in the very initiation of COPD and is closely related to morbidity and mortality. To evaluate the efficacy of Bufei Jianpi granules on SMD, we observed skeletal muscular function and histomorphology, mitochondrial morphormetry and proteins in COPD rats induced by cigarette-smoke and *Klebsiella* pneumoniae.

**Methods:**

Seventy-two Sprague–Dawley rats were randomized into Control + Saline, Control + Bufei Jianpi, Control + Aminophylline, COPD + Saline, COPD + Bufei Jianpi and COPD + Aminophylline groups. From week 9 to 20, rats were administrated intragastricly by normal saline, Bufei Jianpi granules and aminophylline, respectively. Muscular tension and fatigue index of intercostal muscle, quadriceps, biceps and soleus were detected by using electrophysiological technology. Pathological and ultrastructural changes and expressions of mitochondrial Bcl-2 nineteen-kilodalton interacting protein 3 (Bnip3) and cytoplasm cytochrome C (Cyto C) in the four skeletal muscles were observed by using optical and electron microscope and western blotting.

**Results:**

There was no statistical difference among the control rats treated with saline, Bufei Jianpi granules or aminophylline in above-mentioned parameters. Muscular tension, mitochondria volume density (Vv) and compared membrane surface (δm) of the four muscles were significantly lower in COPD + Saline group compared to Control + Saline group, while fatigue index, mitochondria surface area (δ), Bnip3 and Cyto C were higher (P < 0.05). COPD rats showed more morphological changes in muscle tissues than controls, such as atrophy, degeneration, necrosis and matrix hyperplasia. Utrastructurally, mitochondria populations decreased significantly in the four muscles, and were shrunken and even cavitation changed. The up-mentioned parameters were improved in Bufei Jianpi group (P < 0.05) in the four muscles.

**Conclusions:**

Bufei Jianpi granules can improve skeletal muscle function via improving mitochondria population and function, reducing apoptotic factors such as Bnip3 and Cyto C, and is more effective than aminophylline.

## Background

Chronic obstructive pulmonary disease (COPD), a common preventable and treatable disease, is characterized by persistent airflow limitation that is usually progressive and associated with enhanced chronic inflammatory response in the airways and the lung to noxious particles or gases, which also associates with several extrapulmonary manifestations, such as weight loss, malnutrition and skeletal muscle dysfunction (SMD) [1].

As one of the most important extrapulmonary complication of COPD, SMD often appears in the initial phase of the disease and is easily overlooked. Clinically, decline in pulmonary function in patients was generally considered as the main reason for exercise intolerance, but definitely, SMD is a direct precipitating factor which forced COPD patients to stop activities [2]. SMD is also a key factor in accelerating the deterioration of the disease conditions and an independent predictor of morbidity and mortality [3]. Recently, systemic inflammation, skeletal muscle cell apoptosis, energy metabolism disorders, mitochondrial dysfunction, muscular protein synthesis and differentiation/regeneration damage have been involved in the development of SMD, but the exact mechanism remains unclear [4-8].

Tobacco smoking and bacterial infection are the most common and important risk factors for COPD, and have been used to establish animal COPD models [9,10]. *Klebsiella* pneumoniae is one of top three pathogens causing COPD deterioration and community acquired pneumonia [11,12], and has been used to prepare animal models of COPD for both stable and acute exacerbation stages [13]. Previous study indicated that COPD rat induced by cigarette smoke exposures with *Klebsiella* pneumoniae infections is closer to the nature of COPD than classical models [14].

In Traditional Chinese Medicine (TCM), COPD is classified in FEIZHANG and WEIZHENG Disease, which always complicated with SMD. The syndrome of lung-spleen qi deficiency, manifesting shortness of breath and limbs weakness, is a most common syndrome occurring in the very beginning of the disease [15,16]. In TCM, spleen is a very important concept representing the function of digestive system, which can obtain energy (qi) from food and drinks, and strengthen the lung. The dysfunction of the spleen and lung are involved in the pathogenesis, which can directly affect the production and management of qi. Bufei Jianpi granules is a specific prescription for the syndrome of lung-spleen qi deficiency in the treatments of COPD. In previous studies, we found that Bufei Jianpi granules can significantly improve lung function, reduce frequency and extent of acute exacerbations, prolong six-minute walk distance and improve quality of life and have beneficial long-term effects after a 12-month follow-up in COPD patients; and improve pulmonary function and increase diaphragm muscular tension and endurance in cigarette-smoke and *Klebsiella* pneumoniae induced COPD rats [17,18]. Aminophylline is a complex of theophylline and ethylenediamine, which is not only a bronchodilator, but also with pharmacological actions in alleviating inflammatory response and improving the function of diaphragm and peripheral skeletal muscles [19-22].

Mitochondria are the main source of energy for cells. The mechanisms that lead to SMD in COPD have received increasing attention, and mitochondrial dysfunction is recognized as central hub in skeletal muscle deterioration in the progression of the disease [8,23]. Mitochondrial pathophysiology represents an emerging area of research in muscle dysfunction associated with COPD. Skeletal muscle contains a large number of mitochondria, which produced energy by oxidative phosphorylation in physiological conditions. In skeletal muscle tissue, maintaining normal mitochondrial morphology and function is essential for keeping of normal skeletal muscular function.

Bcl-2 nineteen-kilodalton interacting protein 3 (Bnip3) is a pro-apoptotic BH3-only protein associated with mitochondrial dysfunction and cell death and also induces autophagy in cells, and plays a key role in the pathogenesis of many diseases [24-29]. When apoptosis signaling initiated, Bnip3 is translocated to cytoplasm from nucleus and localized on the mitochondrial membrane, and impairs mitochondrial function and induces cell death [24,28]. Cytochrome C (Cyto C) is an important component of the mitochondrial respiratory chain. When mitochondrial function was impaired, Cyto C located in the inner mitochondrial membrane is released into the cytoplasm, and trigger signaling of apoptosis [30]. In this study, we tested the expressions of mitochondrial Bnip3 and cytoplasm Cyto C to explore the function of mitochondria.

To evaluate the efficacy of Bufei Jianpi granules on SMD, comprehensively, in quadriceps, biceps, soleus and intercostal muscles from limbs and thorax, we observed the skeletal muscular function and histomorphology, mitochondrial morphormetry and expressions of impairment related proteins in COPD rats induced by cigarette-smoke and *Klebsiella* pneumoniae.

## Methods

### Animals

Thirty-six male and thirty-six female Sprague–Dawley rats, 2-month-old, weighing 200 ± 20 grams (g), were provided by the Experimental Animal Center of Henan Province (Special Pathogen Free, SCXK (Henan): 2005–0001, Zhengzhou, China) and accommodated in the individual ventilated cases (CA25, Fengshi, Suzhou, China) seven days before experiments in the facility in the First Affiliated Hospital, Henan University of Traditional Medicine, Zhengzhou, Henan, China. Room temperature was maintained at (25 ± 1) °C, relative humidity at (50 ± 10)%, gas changes at 10 ~ 15 times per hour, ammonia concentration less than 14 mg/m^3^, noise ≤ 60 db. Sterilized diet and water were freely accessed.

The protocol of this study was approved by the Ethic Committee of the First Affiliated Hospital, Henan University of Traditional Medicine, Zhengzhou, Henan, China.

### Bacteria

*Klebsiella* pneumoniae (KP; strain No: 46114) provided by the National Center For Medical Culture Collections (Beijing, China), was prepared at a concentration of 6 × 10^8^ colony forming units (CFU) of the suspension liquid before administrated to animals.

### Medicines

Bufei Jianpi granules are main consisted of Huang Qi (*Radix Astragali Mongolici*) 15 g, Dang Shen (*Radix Codonopsis*) 15 g, Bai Zhu (*Rhizoma Atractylodis Macrocephalae*) 12 g, Fu Ling (*Poria*) 12 g, Chuan Bei Mu (*Bulbus Fritillariae Cirrhosae*) 9 g, Di Long (*Pheretima Aspergillum*) 12 g [31]. The Bufei Jianpi granules were prepared and provided by the Department of Pharmacology in the First Affiliated Hospital of Henan University of Traditional Chinese Medicine, Zhengzhou, China. Aminophylline Tablets (0.1 g per tablet) were crashed and prepared into 1 mg · mL^−1^ solution before administrated (Xinhua, Shandong, China).

### Cigarettes

Hongqi Qu® Filter cigarette (tobacco type, tar 10 mg, nicotine content 1.0 mg, carbon monoxide 11 mg) were provided by Henan Zhongyan industrial company (Zhengzhou, Henan).

### Model preparation

After adaptively accommodated to the facility for 7 days, COPD rats were exposed to cigarette smoke and KP for model establishment, while control animals were ventilated with filtered air [14]. Tobacco smoke was generated by a smoke machine (BUXCO, NC, USA), 8 cigarettes per treatment in the first two weeks, and 15 cigarettes per treatment from week 3 through 12, b.i.d. KP solution (0.1 mL) was slowly dropped into the two nostrils in an alternate fashion, per 5 days, for the first 8 weeks [14].

### Grouping and administration

Seventy-two rats were randomly divided into Control + Saline, Control + Bufei Jianpi, Control + Aminophylline, COPD + Saline, COPD + Bufei Jianpi and COPD + Aminophylline groups, using random number table. Six male and six female rats were set in each group. From week 9 through 20, rats were administrated intragastricly by normal saline (2 mL per rat) in Control + Saline and COPD + Saline groups, Bufei Jianpi granules (5 g · kg^−1^ · d^−1^) in Control + Bufei Jianpi and COPD + Bufei Jianpi groups, aminophylline (2.3 mg · kg^−1^ · d^−1^) in Control + Aminophylline and COPD + Aminophylline groups. The equivalent doses of Bufei Jianpi granules and aminophylline were calculated by the following formula according references: D_rat_ = D_human_ × (K_rat_/K_human_) × (W_rat_/W_human_) ^2/3^. D: dose; K: body shape index K = A/W^2/3^ (A: Surface area/m^2^, W: Weight/kg); W: body weight [32].

### Muscular tension and fatigue index

After the rats were anesthetized, second intercostal muscle, quadriceps, biceps and soleus collected from right side thorax and limbs were used for tension and endurance detection. A three-dimensional tissue perfusion system (ADInstruments, Australia), including ML880 Recorder, four-channel bridge amplifier (ML224), four-chamber tissue bath (ML0146/10) and tension sensor (MLT01201) was used for the determinations of muscular tension and fatigue index. The system was calibrated with a 2-g pre-loaded weight. The tissue strips were banded between a needle at the bottom of the bath and the sensor, and immerged into Kreb’s solution ventilated with gas mixed with 95% O2 and 5% CO2. The bath liquid was maintained at 37°C automatically. After balanced for 30 min, the muscle strips were stimulated with 1 Hz, 30 ~ 45 Voltage, 2 ms wavelength square wave, 4 times/min. Five-minute data were recorded and used for calculation of muscular tension (P0).

Muscle fatigue model was induced with a 100 Hz electrical stimulation (wavelength 0.2 ms, 45 Voltage) for 10 min. After stimulated with a 1 Hz, wavelength 2 ms, 30 Voltage wave, 4 times/min, muscular tension (Pt) was collected and averaged. The data were finally corrected by cross sectional area. Fatigue index (fatigue index, FI) = Pt/P0.

### Muscular histomorphology

After rats were sacrificed, second intercostal muscle, quadriceps, biceps and soleus muscles harvested from the left side chest and limbs were transected into three parts, the upper portion for morphological analysis. Formalin fixed intercostal muscle, quadriceps, biceps and soleus tissues were embedded with paraffin and cut into 4 micrometers slices. Deparaffinage and hematoxylin-eosin (HE) staining were performed following the standard operation procedure. All images were taken and observed at amplification of 200 under an Olympus PM-10 AD optical microscope and photographic system (Olympus, Tokyo, Japan).

### Ultrastructure of skeletal muscle cells

After rats were sacrificed, the lower part of the left side second intercostal muscle, quadriceps, biceps and soleus were prepared for ultrastructure observation. Fresh muscles were cut into 1 mm × mm × mm cubes and fixed with 2.5% glutaraldehyde and followed by 1% osmic acid. Ultrathin sectioning was performed after dehydrated in serial ethanol and embedded with Epon-812. Ultrastructural changes were detected using TEM-1400 electron microscope (OLYMPUS, Japan), including mitochondria volume density (Vv), membrane surface (δm) and surface area (δ).

### Western blotting

Intercostal muscle, quadriceps, biceps and soleus were frozen in a −80°C freezer for protein analysis. Skeletal muscles were homogenized on ice in cold RIPA buffer (50 mM Tris–HCl at pH 7.4, 150 mM NaCl, 1% NP40, 0.25% Na-deoxycholate and 1 mM PMSF) with freshly added complete protease inhibitor cocktail. The homogenates were centrifuged for 10 min at 600× g to remove unbroken tissue and nuclei, and the supernatants were centrifuged for 5 min at 3,000× g to pellet mitochondria. The supernatant was further centrifuged for 30 min at 10,000× g to obtain cytosol [28,33]. The mitochondria and cytoplasm protein concentrations were detected by Bradford method and then 2% SDS and 5% 2-mercaptoethanol added before protein denaturalization at 95°C for 5 min. Fifty micrograms of protein was separated by sodium dodecyl sulfate-polyacrylamide gel electrophoresis (SDS-PAGE) and transferred to polyviny lidene difluoride (PVDF) membranes (Millipore, Bedfore, MA, USA). The membranes were blocked with 5% skimmed milk powder in Tris-buffered saline containing 20 mmol/L Tris-buffered saline (pH 7.4), 500 mmol/L NaCl and 0.05% Tween 20, then incubated with primary antibody Bnip3, Cyto C, or β-actin (Santa Cruz, CA, USA) according to instructions and horseradish peroxidase (HRP)-conjugated secondary antibodies (Santa Cruz, CA, USA). Finally, signals were gained by using the Super ECL (enhanced chemiluminescence) Plus reagent (Solarbio, Shanghai, China) and scanned and quantified by PowerLook 2100XL-USB scanner (UMAX, Taiwan) and Image J software (NIH, MD, USA).

### Statistics

Data were analyzed using SPSS 18.0 statistical software. Student-Newman-Keuls method in One-Way ANOVA was used for the detection of difference among groups. All results were expressed as mean ± standard deviation (SD). The significant level was set at α = 0.05.

## Results

### Changes in muscular tension and fatigue index

**Figure 1:**
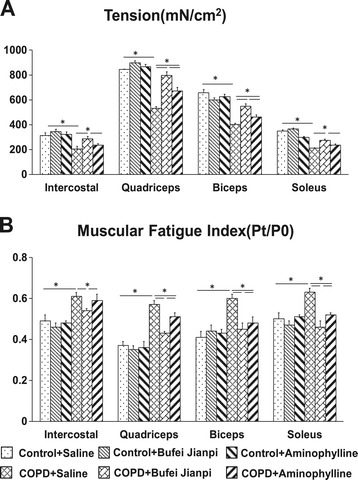
**Changes in muscular tension (A) and fatigue index (B) in intercostal, quadriceps, biceps and soleus muscles in control or chronic obstructive pulmonary disease rats treated with normal saline, Bufei Jianpi granules or aminophylline.** N = 12. *P < 0.05.

As showed in Figure 1, there was no statistical difference in muscular tension and fatigue index in all the four muscles in Control rats treated with saline, Bufei Jianpi or Aminophylline (P > 0.05). Muscular tension of the four muscles decreased significantly in COPD + Saline group compared to Control + Saline group (P < 0.05), while fatigue index increased significantly (P < 0.05). In COPD rats, muscular tension of the four muscles were significantly higher in Bufei Jianpi group than in Saline and Aminophylline groups, while fatigue index were significantly lower in Bufei Jianpi group (P < 0.05). Compare with Saline group, muscular tension of quadriceps and biceps in COPD rats were significantly higher in Aminophylline group, while fatigue index of quadriceps, biceps and soleus were lower (P < 0.05). In the four muscles, quadriceps showed greater muscular tension increase than the other three.

### Morphological changes in skeletal muscles

**Figure 2:**
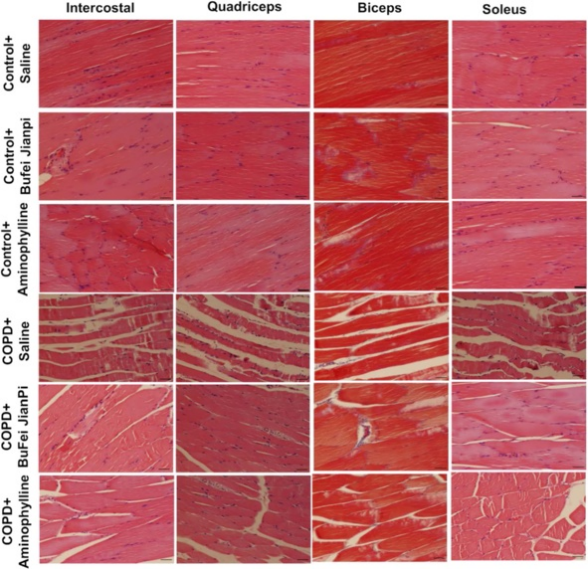
**Representative pathological pictures for intercostal, quadriceps, biceps and soleus muscles in control or chronic obstructive pulmonary disease rats treated with normal saline, Bufei Jianpi granules or aminophylline.** HE stained, amplification × 200. Scale bars: 100 μm.

As showed in Figure 2, no obvious damage was observed in intercostal muscle, quadriceps, biceps or soleus in Control rats treated with saline, Bufei Jianpi or Aminophylline. In COPD rats, there were marked morphological changes in all the four muscles, such as increased interval among muscle fibers, atrophy, degeneration, necrosis and matrix hyperplasia and etc. Compared with Saline-treated COPD rats, the up-mentioned changes were improved in Bufei Jianpi and Aminophylline groups, especially in Bufei Jianpi group.

### Ultrastructural changes in skeletal muscle tissues

**Figure 3:**
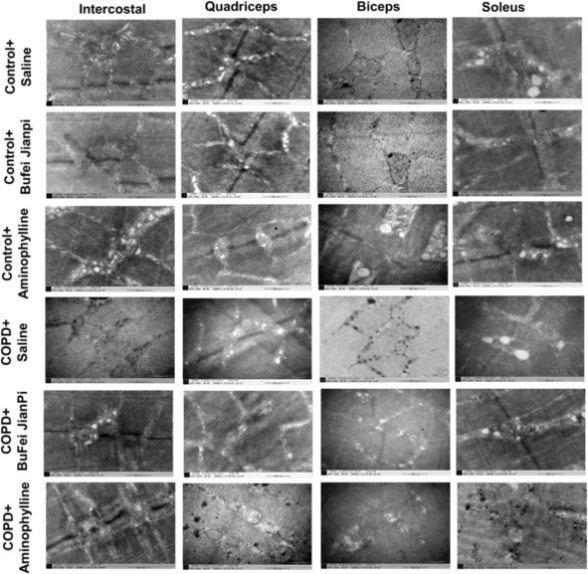
**Representative ultrastructural changes pictures of intercostal, quadriceps, biceps and soleus muscles in control or chronic obstructive pulmonary disease rats treated with normal saline, Bufei Jianpi granules or aminophylline.** Amplification × 50000.

As shown in Figure 3, there was no marked mitochondrial impairment in the four muscles in control rats treated with saline, Bufei Jianpi or Aminophylline. In COPD rats, mitochondria reduced in population and shrunken in the four muscles, mitochondrial cristae disappeared and even cavitation changed. Compared with COPD rats treated with saline, the conditions of mitochondria were improved in Bufei Jianpi and aminophylline groups, especially in Bufei Jianpi group.

**Figure 4:**
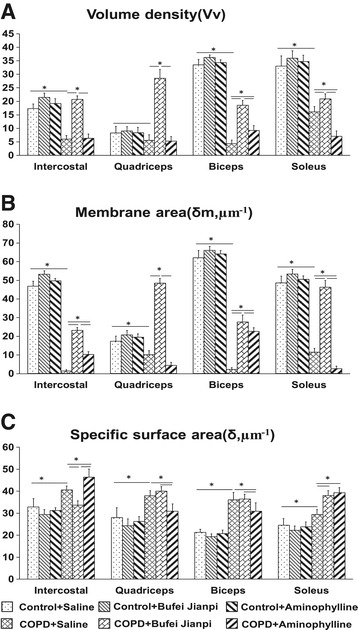
**Changes of mitochondria volume density (Vv) (A), ratio of membrane area (δm) (B) and specific surface area (δ) (C) in intercostal, quadriceps, biceps and soleus muscles in control or chronic obstructive pulmonary disease rats treated with normal saline, Bufei Jianpi granules or aminophylline.** Vv, δm is positive correlated to the function of mitochondria. δ is negative correlated to the activity of mitochondria. N = 6. *P < 0.05.

As shown in Figure 4, there was no difference observed in Vv, δm or δ in the four muscles in control rats treated with saline, Bufei Jianpi or aminophylline (P > 0.05). However, Vv, δm in the four muscles decreased significantly in COPD + Saline group compared to Control + Saline group (P < 0.05), while δ increased in COPD + Saline group (P < 0.05). In COPD rats, Vv and δm were significantly higher in the four muscles in Bufei Jianpi group than in saline and aminophylline groups, while δ of intercostal muscle was lower (P < 0.05). Vv and δm of biceps and soleus, δm of intercostal muscle in aminophylline group were higher than in saline group, while δ of quadriceps and biceps was lower (P < 0.05).

### Expressions of Bnip3 and Cyto C in skeletal muscle tissues

**Figure 5:**
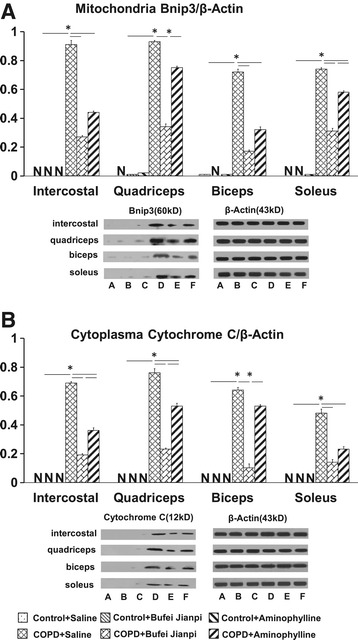
**Expressions of mitochondrial Bnip3 (A) and cytoplasm cytochrome C (B) in intercostal, quadriceps, biceps and soleus muscles in control or chronic obstructive pulmonary disease rats treated with normal saline, Bufei Jianpi granules or aminophylline.** N = 6. *P < 0.05. A: Control + Saline, B: Control + Bufei Jianpi, C: Control + Aminophylline, D: COPD + Saline, E: COPD + Bufei Jianpi, F: COPD + Aminophylline. N in Figure **A** and **B** represents NOT DETECTED or the value of Bnip3/β-actin or cytochrome C/β-actin APPROXIMATES TO ZERO.

As shown in Figure 5, there was no difference in mitochondrial Bnip3 and cytoplasm Cyto C in the four muscles in control rats treated with saline, Bufei Jianpi granules or aminophylline. Bnip3 and Cyto C increased significantly in the four muscles in COPD + Saline group compared to Control + Saline group (P < 0.5). In COPD rats treated with Bufei Jianpi granules, Bnip3 and Cyto C in the four muscles were significantly lower than in saline-treated group (P < 0.05). Bnip3 in the quadriceps, soleus and Cyto C in intercostal muscle, quadriceps and biceps were significantly lower in Bufei Jianpi group than in aminophylline group (P < 0.05). Bnip3 in the intercostal muscle, biceps, soleus and Cyto C in intercostal muscle, quadriceps and soleus were significantly lower in aminophylline group than in saline-treated group (P < 0.05).

## Discussion

This is the first study to explore the beneficial effects of Bufei Jianpi granules on skeletal muscle and mitochondrial dysfunction (SMD) in a COPD rat model induced by cigarette-smoke and bacterial infections exposures. In this study, we found Bufei Jianpi granules can improve skeletal muscle function via improving mitochondrial morphormetry and function.

SMD often occurs in the early course of the disease and is a key factor which severely affects patients’ exercise tolerance leading to disability and poor quality of life. SMD has been associated with frequent exacerbations, a reduced health status and a higher mortality rate, independently of disease [34,35]. Recently, more and more attention has been focused to COPD patients complicated with SMD, but how to slow down the progression of SMD has become a key point to reverse COPD.

In Traditional Chinese Medicine (TCM), COPD patients complicated with SMD belongs to the category of FEIZHANG and WEIZHENG. Spleen qi, a unique concept in TCM, which represents the function of digestive system, can digest, obtain and transfer energy (qi) from food and drinks, and strengthen the lung qi. The dysfunction of the spleen and lung are involved in the pathogenesis of COPD, which can directly affect the production and management of qi. Previous studies have shown that deficiency of lung-spleen qi is one of the most common syndromes in stable COPD [15,16]. Bufei Jianpi granules is a specific prescription for this syndrome, which has been proved effective in improving lung function, reducing frequency and duration of acute exacerbation, prolonging six-minute walk distance and improving quality of life in COPD patients, and have beneficial long-term effects after a 12-month follow-up [17]. In experimental studies, we found that Bufei Jianpi granules can improve pulmonary function and diaphragmatic muscle tension and endurance, and alleviate pulmonary and systemic inflammation in COPD rats [18,32].

The occurrence of SMD in COPD patients often leads to the impairments of exercise ability and even daily activities. Previously, the activity reduction of COPD patients was attributed to the decline of pulmonary function, but recently, more and more evidences suggest that SMD has become a key factor of impeding daily activities of COPD patients and is an important factor in inducing exacerbations, which can seriously affect the patients’ quality of life and greatly increase morbidity and mortality [2,3]. In the general population, quadriceps weakness has been used as a predictor of mortality [36]. Studies have shown that muscular tension of quadriceps declined in about one-third population of COPD patients [37]. Quadriceps asthenia is closely related to peripheral muscle fiber morphological changes, volume and mass reduction and the mortality of COPD patients, which can be used as prognostic information that provided by age, body mass index (BMI) and forced expiratory volume in 1 second (FEV1) [38]. Skeletal muscle tension is largely determined by the condition of muscle fibers. Muscle fiber thinning will directly lead to skeletal muscle atrophy and decreased muscular tension. In this study, obvious atrophy characterized by disorganized muscle fibers and widened gaps among fibers was found in intercostal muscle, quadriceps, biceps and soleus in COPD rats induced by cigarette-smoke and bacterial exposures, as well as the muscular tension and endurance declined. Bufei Jianpi granules can significantly improve the histomorphology, muscular tension and endurance in peripheral striated muscles, especially in intercostal muscle and quadriceps, and shows more benefit than aminophylline.

Mitochondria are the most important organelles in cells, which reflect the metabolic level of the cells. Generally, mitochondria are the main source of energy for cells and are involved in production of reactive oxygen species and activation of apoptosis [39]. Numerous aspects of mitochondrial function have been found impaired in skeletal muscle in COPD patients, mainly in vastus lateralis, which is characterized by decreased mitochondrial density, impaired activity and coupling of mitochondrial respiratory chain complexes, increased mitochondrial production of reactive oxygen species and, possibly, increased apoptosis [8,40]. Usually, Vv, δ and δm are the most common parameters to represent population and morphology of mitochondria, which can indirectly reflect mitochondrial function. Vv is the total volume of mitochondria per unit volume, which is positive correlated to the number and function of mitochondria. δ is the degree of mitochondrial swelling, which is negative correlated to the activity of mitochondria. δm is an indicator of mitochondrial membrane changing and cristae membrane area changing, and also considered positive correlated to the metabolic activity of cells. Our data show that mitochondrial population and function decreased significantly in intercostal muscle, quadriceps, biceps and soleus in COPD rats. Compared to saline and aminophylline groups, Bufei Jianpi granules can significantly improve Vv and δm in four muscles in COPD rats, while reduce δ in intercostal muscle, and shows more benefit.

Mitochondrial dysfunction is the main course and character of cell death, which can lead apoptosis and autophagy [41]. It can be physiological but can also lead to diseases, including skeletal muscle atrophy/dysfunction [23,28]. Cell apoptosis and autophagy are important causes of mitochondrial dysfunction. Cyto C and Bnip3 play very important roles in apoptosis and autophagy. Normally, Bnip3 localizes in the nucleus. When hypoxia or oxidative stress happened, Bnip3 transfers to cytoplasm and localizes to the mitochondria membrane, where it induces mitochondrial dysfunction and subsequent cell death and also induces autophagy in cells [28,42]. Generally, Cyto C locates in mitochondria gap between the outer and inner membranes. When mitochondrial function is affected and membrane permeability increased, Cyto C will be released into cytoplasm, and then caspase-3 and other signaling pathway of apoptosis will be activated [30,43]. In this study, we found mitochondrial Bnip3 and cytoplasm Cyto C increased significantly in COPD rats, and were reduced by Bufei Jianpi granules and Aminophylline, especially Bufei Jianpi granules. It indicates that Bufei Jianpi granules can improve peripheral skeletal muscle function via improving mitochondrial function, reducing the release of apoptotic and autophagy factors. This result might be involved in the mechanism of how Bufei Jianpi granules improve exercise ability and tolerance, and even pulmonary function in COPD patients, which is consistent with previous data [17,18].

## Conclusions

In conclusion, Bufei Jianpi granules can improve skeletal muscle function via improving mitochondria population, morphormetry and function, reducing release of apoptotic factors such as Bnip3 and Cyto C, and is more effective than aminophylline.
